# Metabolism and physiology of pathogenic bacterial obligate intracellular parasites

**DOI:** 10.3389/fcimb.2024.1284701

**Published:** 2024-03-22

**Authors:** Cameron G. Mandel, Savannah E. Sanchez, Colleen C. Monahan, Weerawat Phuklia, Anders Omsland

**Affiliations:** ^1^ Paul G. Allen School for Global Health, College of Veterinary Medicine, Washington State University, Pullman, WA, United States; ^2^ Department of Microbiology and Immunology, Virginia Commonwealth University School of Medicine, Richmond, VA, United States; ^3^ Lao-Oxford-Mahosot Hospital-Wellcome Trust Research Unit, Microbiology Laboratory, Mahosot Hospital, Vientiane, Lao People’s Democratic Republic

**Keywords:** obligate, nutrient, genome streamlining, physiology, metabolism, auxotrophy

## Abstract

Bacterial obligate intracellular parasites (BOIPs) represent an exclusive group of bacterial pathogens that all depend on invasion of a eukaryotic host cell to reproduce. BOIPs are characterized by extensive adaptation to their respective replication niches, regardless of whether they replicate within the host cell cytoplasm or within specialized replication vacuoles. Genome reduction is also a hallmark of BOIPs that likely reflects streamlining of metabolic processes to reduce the need for *de novo* biosynthesis of energetically costly metabolic intermediates. Despite shared characteristics in lifestyle, BOIPs show considerable diversity in nutrient requirements, metabolic capabilities, and general physiology. In this review, we compare metabolic and physiological processes of prominent pathogenic BOIPs with special emphasis on carbon, energy, and amino acid metabolism. Recent advances are discussed in the context of historical views and opportunities for discovery.

## Introduction

### Bacterial obligate intracellular parasites

**Figure 1:**
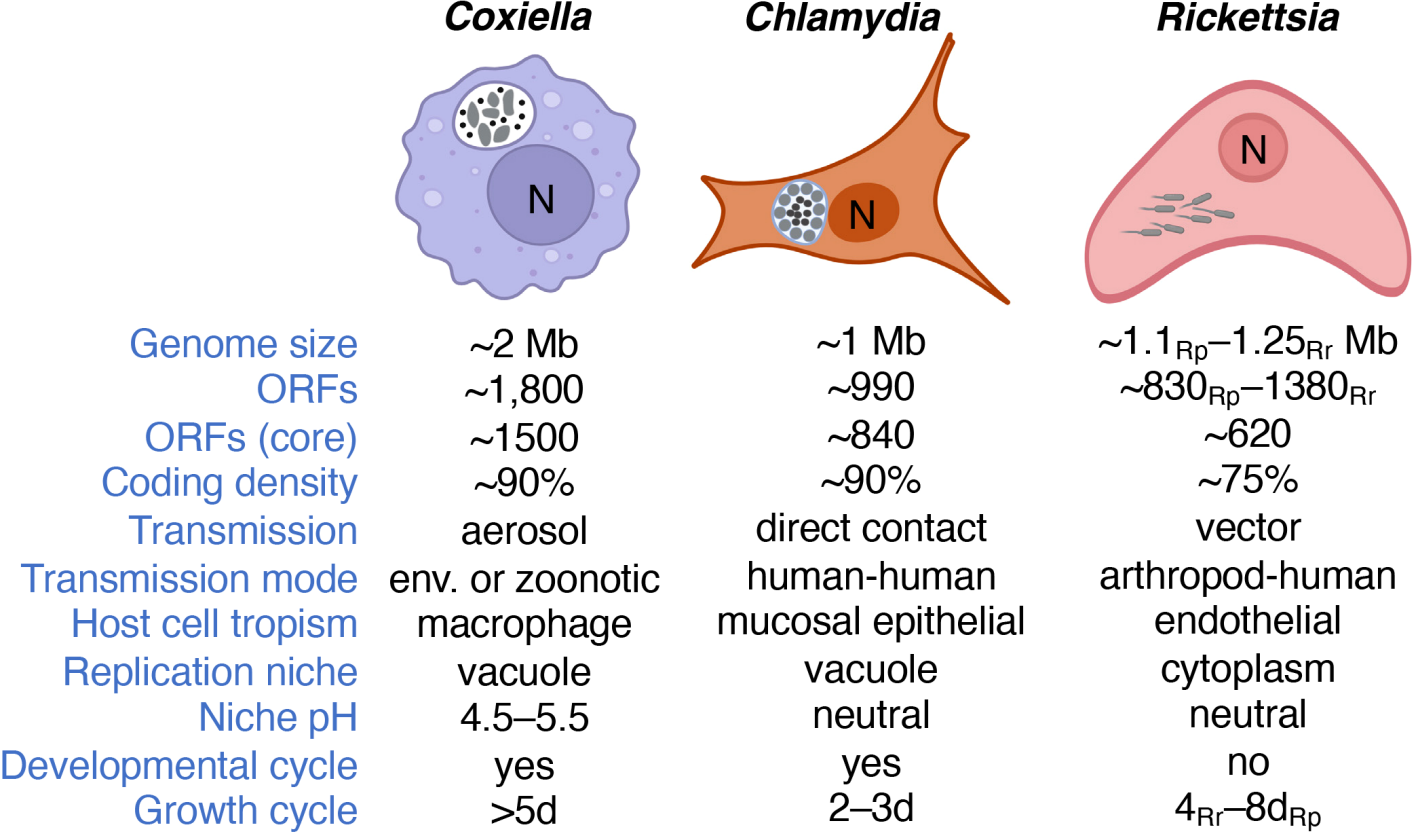
Major characteristics and intracellular lifestyles of *Coxiella*, *Chlamydia* and *Rickettsia.* Information is based on *C. burnetii*, *C. trachomatis*, and *R. rickettsii* unless otherwise specified. Note that differences in genome size, gene content, and/or arthropod vector exist between species of the same genus. *C. burnetii* is depicted as characteristic pleomorphic cells including both SCVs and LCVs. *C. trachomatis* is depicted with both EBs and RBs, the latter lining the inner inclusion membrane. *R. rickettsii* is illustrated with the characteristic actin tails that allow motility. We note that only the primary host cell type is indicated and that *C. burnetii* also shows tropism for placental trophoblasts. The number of ORFs refers to that of *C. burnetii*, *C. trachomatis*, *R. prowazekii* (Rp) and *R. rickettsii* (Rr), or the core genomes for the genus. *R. prowazekii* and *R. rickettsii* represent the typhus and spotted fever groups of this genus, respectively. Growth cycle refers to the approximate time required to reach stationary phase during infection of cultured Vero cells. Bacteria are shown in gray. “N” denotes the host cell nucleus and “env.” environmental (e.g., contaminated soil).

Bacterial obligate intracellular parasites (BOIPs) represent a unique group of bacteria that all depend on invasion of a eukaryotic host cell to reproduce (Casadevall, 2008). BOIPs are true parasites due to their negative impacts on the host cell, including opportunistic scavenging of host cell-synthesized nutrients, and active establishment and maintenance of unique replication vacuoles for some of these pathogens. We base the designation of an obligate intracellular lifestyle solely on information from the natural life cycle of the pathogen and not on the possibility of host cell-free (i.e., axenic) culture in a laboratory setting. As such, *Coxiella burnetii* remains a BOIP despite the possibility of culturing this organism axenically (Omsland et al., 2009). Prominent pathogenic BOIPs include species of the genera *Coxiella*, *Chlamydia*, *Rickettsia*, *Anaplasma*, *Ehrlichia*, and *Orientia*. This review will focus on the first three genera, which collectively represent variations in the obligate intracellular lifestyle and BOIP-host interaction (**Figure 1**). Reference will be made to BOIPs of other genera when appropriate.

#### Coxiella

The discovery of *C. burnetii* appeared co-incident with an outbreak of a new febrile illness (Burnet and Freeman, 1937; Derrick, 1937), later to be called Query (Q) fever, in slaughter-house workers in Australia. Interestingly, a team of American scientists isolated *C. burnetii* from ticks around the same time (Davis and Cox, 1938). The pathogen has since been recognized as a major zoonotic bacterium most notably related to infection of agricultural animals, including goats (Schneeberger et al., 2014). *C. burnetii* is a moderate acidophile adapted to replicate within a phagolysosome-derived vacuole termed the *Coxiella* Containing Vacuole (CCV) (Coleman et al., 2004; Voth and Heinzen, 2007; Kohler and Roy, 2015). *C. burnetii* transitions between two cell forms, the replicative Large Cell Variant (LCV) and the non-replicative Small Cell Variant (SCV) that accumulates in stationary phase (Coleman et al., 2004). Both the LCV and SCV forms of *C. burnetii* can establish infection in cultured cells (Coleman et al., 2004; Sandoz et al., 2014).

#### Chlamydia

Different species and pathotypes (aka, serovars) of the *Chlamydia* genus can cause a range of diseases including sexually transmitted infections, the blinding condition trachoma, as well as respiratory infections (Mishori et al., 2012; Lane and Decker, 2016; Porritt and Crother, 2016). Like *Coxiella*, *Chlamydia* species can cause disease in animals other than humans (Roulis et al., 2013; Borel et al., 2018). Also, *Chlamydia* species, including *C. trachomatis* and *C. pneumoniae*, replicate within a vacuole in the host cell cytoplasm termed the chlamydial inclusion (Moore and Ouellette, 2014). Development of the chlamydial inclusion depends on bacterial protein synthesis but not pathogen replication (Engström et al., 2015). Because the chlamydial inclusion does not fuse with lysosomes, the inclusion, unlike the CCV, is a non-degradative compartment with neutral pH (Heinzen et al., 1996). Members of the *Chlamydia* genus transition between the infectious but non-replicative Elementary Body (EB) and replicative but non-infectious Reticulate Body (RB) (Shaw et al., 2000; Belland et al., 2003b).

#### Rickettsia

Pathogens in the *Rickettsia* genus are arthropod-borne bacteria associated with febrile illness that can cover a wide symptomatic range, including acute, chronic, and reoccurring disease (Abdad et al., 2018; Blanton, 2019). *R. prowazekii* and *R. rickettsii*, the agents of epidemic typhus and Rocky Mountain Spotted Fever, respectively, replicate within the host cell cytosol. Spotted fever group *Rickettsia*, including *R. rickettsii*, utilize actin-based motility to spread directly from an infected host cell to an adjacent non-infected cell (Teysseire et al., 1992; Heinzen et al., 1993). Members of the typhus group *Rickettsia*, including *R. prowazekii*, are non-motile or produce short actin tails (Heinzen et al., 1993). Bacteria in the *Rickettsia* genus can be transmitted by various arthropod vectors including lice, fleas, mites, and ticks and establish disease in various animal species (Walker and Ismail, 2008).

Neither *C. burnetii* nor pathogenic *Chlamydia* species are motile. Moreover, species of the genus *Rickettsia* do not transition between cell forms.

## Diversity in metabolic capacity of BOIPs

Although an understanding of the metabolic capabilities exhibited by *C. burnetii*, *C. trachomatis* and *R. prowazekii* saw tremendous progress during the pre-genomic era, it seems fair to say that a real sense of each pathogen’s metabolic capacity was not appreciated until their genome sequences were used as a basis for metabolic pathway reconstruction between 1998 and 2003 (Andersson et al., 1998; Stephens et al., 1998; Seshadri et al., 2003). Indeed, shortly after their discoveries, researchers questioned whether many of the pathogens known today as BOIPs were bacteria or viruses (e.g., (Ormsbee and Peacock, 1964). Early descriptions of metabolic and physiological features of these organisms were likely heavily influenced by an expectation of limited autonomous metabolic capacity. Moreover, depending on context, intracellular replication niches have been described as either nutrient rich or hostile to invasive bacteria (Moulder, 1974; Kaufmann, 2011), consistent with significant adaptability to reside intracellularly. With a relatively short history of research and severe technical challenges related to axenic culture and genetic manipulation, current understanding of BOIP metabolism and physiology remains limited. At the same time, the many knowledge gaps that exist makes this a field of research poised for discovery.

### Energy and central metabolism

**Figure 2:**
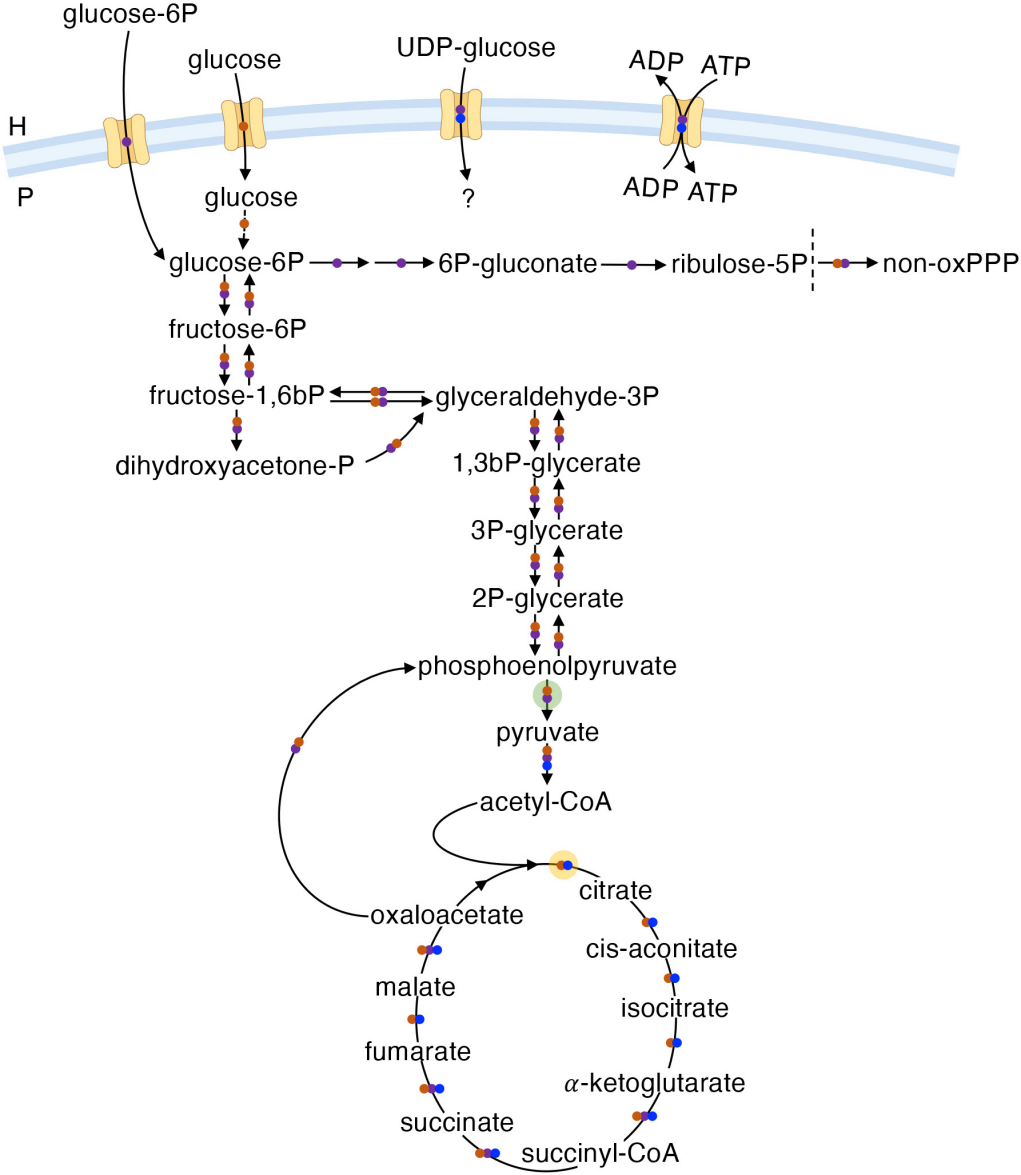
The central metabolic machinery. Overview of central metabolism in *C. burnetii* (RSA493) (●), *C. trachomatis* (Bu/434) (●), and *R. prowazekii* (Madrid E) (●). H and P denote host cell or pathogen, respectively. The broken vertical line separates the oxidative and non-oxidative branches of the PPP. The mechanism for glucose phosphorylation in *C. burnetii* is unknown. The question mark for UDP-glucose indicates potential use in various reactions. Transparent discs indicate bacterial enzymes with regulatory characteristics of eukaryotic proteins in *Coxiella* (yellow) or *Chlamydia* (green).

BOIPs display remarkable differences in central carbon and energy metabolism; *Coxiella*, *Chlamydia*, and *Rickettsia* serve as good representatives of this diversity (**Figure 2**). Central/core metabolism encompasses the glycolytic/gluconeogenic pathways, pentose phosphate pathway (PPP), and the tricarboxylic acid (TCA) cycle; the pathways from which core metabolites are derived (Noor et al., 2010). The general structure of the central metabolic machinery differs between *Coxiella* and *Chlamydia* in that the former has lost the oxidative branch of the PPP while the latter has lost a significant portion of the TCA cycle with the genes encoding citrate synthase, aconitase, and isocitrate dehydrogenase missing (Stephens et al., 1998). Of likely significance to the metabolic capacity and plasticity between chlamydial serovars, some strains (e.g., L2/434/Bu and L2/UCH-1/proctitis) show additional TCA cycle abnormalities at the genome level (Thomson et al., 2008; Omsland et al., 2014). While both *Chlamydia* (Tjaden et al., 1999) and *Rickettsia* (Audia and Winkler, 2006) can scavenge ATP from the host via ATP/ADP translocases, *Coxiella* does not rely on energy parasitism. The oxidation of glucose via glycolysis or the PPP in *Coxiella* (Esquerra et al., 2017) and *Chlamydia* (Schwöppe et al., 2002; Omsland et al., 2012) is dependent on the availability of either non-phosphorylated or phosphorylated forms of glucose, respectively. Conversely, enzymes for glycolysis/gluconeogenesis and the PPP are not encoded by species of the genus *Rickettsia* (Driscoll et al., 2017). In *R. prowazekii*, acquisition of “glucose” as a biochemical moiety for biosynthetic purposes is achieved via transport of uridine 5’-diphosphoglucose (UDP-glucose) rather than glucose itself (Winkler and Daugherty, 1986). In *Chlamydia*, UDP-glucose is brought into the inclusion via the host SLC35D2 transporter and used as a substrate in pathogen-driven glycogen synthesis (Gehre et al., 2016).

As illustrated in **Figure 2**, the genomes of both *Coxiella* and *Chlamydia* encode central metabolic enzymes with regulatory characteristics of their eukaryotic counterparts. For instance, citrate synthase of *C. burnetii* is inhibited by ATP (Heinzen and Mallavia, 1987), while pyruvate kinase from *C. trachomatis* is inhibited by AMP, GTP and ATP but activated by host cell-derived fructose-2,6-bisphosphate (Iliffe-Lee and McClarty, 2002). The expression of enzymes in BOIPs with regulatory characteristics akin to eukaryotic proteins may reflect adaptation to the chemical environment of the eukaryotic host cell. Moreover, these enzymes could serve as a mechanism to interconnect pathogen physiology with the physiological state of the host. Interestingly, host cell-derived pyruvate kinase (as well as aldolase A and lactate dehydrogenase) is detected at the chlamydial inclusion membrane and depletion of (host) aldolase A results in decreases in inclusion size and infectious EB progeny (Ende and Derré, 2020). The presence of host glycolytic enzymes in proximity to the chlamydial inclusion could serve to supply the pathogen and/or reactions governing the inclusion-host interaction with specific and critical glycolytic intermediates (Ende and Derré, 2020).

Early studies with *C. burnetii* under axenic conditions at neutral pH pointed to limited ability of this organism to metabolize autonomously. Nevertheless, some enzymatic activities were detected in bacterial extracts, including that of glycolytic enzymes (Paretsky et al., 1958, 1962; Ormsbee and Peacock, 1964; McDonald and Mallavia, 1970, 1971). The discovery that *C. burnetii* colocalizes with lysosomal enzymes within CCVs (Burton et al., 1971; Burton et al., 1978), combined with knowledge of the acidic pH of phagolysosomes (Ohkuma and Poole, 1978), suggested *C. burnetii* metabolism is strictly pH-dependent. Accordingly, Hackstadt and Williams demonstrated *C. burnetii* reliance on moderately acidic pH for metabolic activation and catabolism of both glucose and glutamate (Hackstadt and Williams, 1981a). Shortly thereafter, glutamate was identified as the preferred energy source of *C. burnetii* (Hackstadt and Williams, 1981b).

Data in support of glucose utilization by *C. burnetii* dates to the 1960s when “hexokinase activity” was demonstrated in bacterial cytoplasmic extracts (Paretsky et al., 1962). More recently, using a *C. burnetii* mutant unable to undergo gluconeogenesis due to deletion of phosphoenolpyruvate carboxykinase (PEPCK, encoded by *pckA*, the first committed step of gluconeogenesis) in combination with the chemically defined medium D-ACM, glucose utilization by *C. burnetii* for biomass production has been confirmed to be nearly as efficient as growth on amino acids (Esquerra et al., 2017). Indeed, *C. burnetii* can acquire glucose by at least two transporters (Kuba et al., 2019). The mechanistic redundancy revealed by the ability of *C. burnetii* to take up glucose via more than one transporter (Kuba et al., 2019) suggests glucose is critical for the metabolic fitness of this organism. Data from genome-wide transcriptional analysis of *C. burnetii* during infection of mice actually indicates that glucose is the principal carbon source while fatty acids are used for energy metabolism (Kuley et al., 2015). Preferential utilization of glucose rather than amino acids by *C. burnetii* to drive central metabolism contradicts earlier interpretations that were based on analysis of energy charge and ATP pool stability, rather than generation of biomass. Importantly, the ability of any pathogen to use a wide variety of substrates in energy metabolism is more relevant than the identification of a “preferred” substrate, the nature of which likely depends on the type of tissue colonized within infected animals as well as the physiological state of the animal. As for *C. burnetii*, PEPCK is also encoded by the *C. trachomatis* genome. Because generation of *C. trachomatis* EBs and relative ATP pools are reduced in host cells cultured with gluconeogenic substrates compared to host cells cultured with excess glucose (Iliffe-Lee and McClarty, 2000), gluconeogenic capacity could be interpreted to have limited significance for *C. trachomatis* EB generation. Additionally, absence of a prototypical fructose 1,6-bisphosphatase (EC 3.1.3.11) could further reduce gluconeogenic capacity in *C. trachomatis* (Mehlitz et al., 2016). However, because *pckA* has been retained and is expressed maximally by replicating RBs (Skipp et al., 2016), and alternative enzymes encoded by *C. trachomatis* (e.g., EC 2.7.1.11 and 2.7.1.90) could serve to convert fructose 1,6-bisphosphate to fructose-6P, gluconeogenic capacity may affect replication and/or EB generation under specific, yet undetermined, conditions. Proteomic analyses have provided somewhat different pictures regarding the expression of central metabolic enzymes, including glycolytic enzymes, in EBs and RBs (Saka et al., 2011; Skipp et al., 2016). Regardless, current data point to a significant role for glycolysis in the EB form.

Emilio Weiss and colleagues described oxidation of glucose (Weiss et al., 1964) and utilization of glucose-6P or glucose in the presence of ATP (Weiss, 1965; Weiss and Wilson, 1969) by *Chlamydia*, also during the 1960s. In *C. trachomatis*, glucose metabolism is mechanistically and physiologically different from that observed in *C. burnetii*. First, while *C. burnetii* can acquire non-phosphorylated glucose (Hackstadt and Williams, 1981a; Esquerra et al., 2017; Kuba et al., 2019), *C. trachomatis* appears dependent on glucose-6P (Omsland et al., 2012; Gehre et al., 2016), acquired via the UhpC transporter (McClarty, 1999; Schwöppe et al., 2002). Because *C. trachomatis* acquires glucose-6P from the host rather than expending ATP for its phosphorylation, and because pyrophosphate (PPi) is used in formation of fructose 1,6-bisphosphate, *C. trachomatis* has been postulated to gain a net 4 ATP molecules rather than 2 during glycolytic activity (McClarty, 1999). The physiological impact of impaired glucose metabolism in *C. trachomatis* has been illustrated by incubation with KSK120, a compound that inhibits uptake and utilization of glucose-6P in this organism (Engström et al., 2014). Similar to the observed loss of infectivity upon depriving *Chlamydia*-infected host cells for glucose (Harper et al., 2000; Iliffe-Lee and McClarty, 2000), treatment of *C. trachomatis*-infected HeLa cells with KSK120 during infection reduced generation of infectious EB progeny. Thus, metabolism of glucose-6P appears to be critical for re-generation of EBs following reproduction via RB replication. While not universally relevant to *Chlamydia* species, loss of GlgA (glycogen synthase) activity results in impaired infectivity in *C. muridarum* (Gehre et al., 2016), again connecting metabolism of glucose to chlamydial virulence. Utilization of glucose-6P may be more significant for protein synthesis in EBs compared to RBs (Omsland et al., 2012), the latter of which has been shown to behave as an energy parasite by scavenging ATP, as well as other NTPs, from the host cell (Tipples and McClarty, 1993). Beyond a critical role for generation of infectious EBs, metabolism of glucose enhances infectivity of *Protochlamydia amoebophila* (Sixt et al., 2013). Glucose metabolism, including oxidation of glucose-6P, also enhances EB metabolism and has been suggested to underlie maintenance of EB infectivity in extracellular environments by both pathogenic and non-pathogenic *Chlamydia* species (Omsland et al., 2012; Sixt et al., 2013; Grieshaber et al., 2018). Utilization of glucose or glucose-6P in *Coxiella* and *Chlamydia* stand in stark contrast to current understanding of central carbon metabolism in *Rickettsia*. In fact, a general lack of genes related to glycolysis and gluconeogenesis in *Rickettsia* (Driscoll et al., 2017) is consistent with a limited role for glucose metabolism in these organisms and an absolute requirement to obtain relevant core metabolites from the host cell.

Following phosphorylation of glucose to glucose-6P, this metabolite is generally destined to be processed via one of two distinct paths in the cell, namely glycolysis or the PPP. The PPP is further divided into two branches: an oxidative branch (oxPPP), considered a major source for recovery of the reducing equivalent NADPH; and a non-oxidative branch (non-oxPPP), critical for synthesis of ribose-5P, which is ultimately required for the biosynthesis of nucleic acid precursors, or erythrose-4P, a precursor for generation of aromatic amino acids. *C. burnetii* lacks two enzymes of the oxPPP (glucose-6P dehydrogenase and 6-phosphogluconate dehydrogenase), suggesting negative selective pressure on this pathway in *C. burnetii*. Given the apparent significance of NADPH, what selective pressure would cause *C. burnetii* to lose the oxPPP? In a recent study, *C. burnetii* was transformed with the gene encoding glucose-6P dehydrogenase, *zwf*, to address this question (Sanchez and Omsland, 2021). While *C. burnetii* expressing *zwf* behaved similarly to the parental strain under glucose excess, the transformant showed significantly reduced ability to replicate under glucose limitation. Expression of *zwf* also resulted in impaired pathogen intracellular replication in J774A.1 cells but not in Vero cells, suggesting some cell types represent a glucose-limiting environment for *C. burnetii*. Impaired replication under glucose-limiting conditions therefore provides some explanation for why *C. burnetii* has lost oxPPP capacity. Identification of the NADPH-regenerating enzyme SdrA (Bitew et al., 2020) suggests *C. burnetii* has evolved to generate NADPH via mechanisms complimentary to the oxPPP, possibly to circumvent metabolic conflicts under low glucose availability.

Bovarnick and Schneider demonstrated over 60 years ago that both ATP generated endogenously by *R. prowazekii* and ATP supplemented to the medium were necessary to stimulate axenic protein synthesis in this organism (Bovarnick and Schneider, 1960). The ATP/ADP translocase utilized by *Rickettsia* for ATP acquisition has since been characterized in detail (Audia and Winkler, 2006). There is also evidence for the capacity of *Rickettsia* species to use glutamate as an energy source (Bovarnick and Miller, 1950; Rees and Weiss, 1968; Williams and Weiss, 1978). Despite the capacity to scavenge ATP from the host cell, recent data suggest that *C. trachomatis* also relies on ATP generated via the combined activities of the pathogen’s sodium pump (Na^+^-NQR) (Dibrov et al., 2004) and a Na^+^-permissive A_1_-A_0_-ATPase during the replicative phase of the chlamydial developmental cycle (Liang et al., 2018). The ability of *C. trachomatis* to generate ATP via a sodium gradient and reliance of the energy thus generated for replication adds another dimension to the energetics of chlamydial metabolism and further challenges the “energy parasite” hypothesis proposed for this organism in the 1960s–70s (Moulder, 1962, 1974), stating that the pathogen depends on host-derived ATP. Because *Chlamydia*-infected host cells require ATP for viability, axenic culture will likely be necessary to resolve questions about the degree to which these pathogens depend on extracellular ATP.

Aspects of central metabolic activity in *Rickettsia* can also take place via atypical mechanisms. In *R. prowazekii*, sn-glycerol-3-phosphate needed for biosynthesis of phospholipids is acquired by importing dihydroxyacetone phosphate (DHAP) with subsequent conversion to sn-glycerol-3-phosphate by GpsA, a glycerol-3P dehydrogenase (G3PDH) (Frohlich et al., 2010). Typically, G3PDH is an enzyme integrated with activities of glycolysis and gluconeogenesis as the substrate DHAP is an intermediate of these pathways. Interestingly, despite lack of genes for complete glycolytic/gluconeogenic pathways, *R. prowazekii* has retained sn-glycerol-3-phosphate dehydrogenase to convert DHAP to G3P to support phospholipid biosynthesis (Frohlich et al., 2010). As noted by Frohlich and colleagues, this novel mechanism can explain the evolutionary pressure to retain G3PDH in *Rickettsia*.

Major distinguishing metabolic features of *Coxiella*, *Chlamydia* and *Rickettsia* are listed in **Table 1**.

**Table 1:** Major Distinguishing Metabolic Features of *Coxiella*, *Chlamydia* and *Rickettsia.*

Feature		*Coxiella*	*Chlamydia*	*Rickettsia*
Carbon source(s)^1^	A	glucose amino acids, inc. glutamate	glucose-6P α-ketoglutarate	UDP-glucose amino acids, inc. glutamine
	B			
Energy source(s)^2^	C	glucose amino acids, inc. glutamate TCA cycle intermediates N/A	glucose-6P N/A α-ketoglutarate host-derived ATP	N/A glutamate N/A host-derived ATP
	D			
	E			
	F			
Mechanism(s) of energy generation or acquisition	G	substrate level phosphorylation oxidative phosphorylation N/A	substrate level phosphorylation oxidative phosphorylation ATP scavenging from host cell	N/A oxidative phosphorylation ATP scavenging from host cell
	H			
	I			
pH		moderate acidophile	neutrophile	neutrophile
O_2_^3^		microaerophile	uncertain	uncertain
CO_2_^4^		required	uncertain	uncertain

^1,2^Information is limited to the expected main substrates and mechanisms.

^3,4^Requirements for O_2_ and CO_2_ is based on optimal growth conditions. Inability to culture *Chlamydia* and *Rickettsia* species under axenic conditions complicates direct analysis of the effect of O_2_ and CO_2_ on these organisms.

Letter codes denote substrate type or mechanism: A and C – glucose or glucose derivatives; B – gluconeogenic substrates; D – amino acids; E – TCA cycle intermediates; F – ATP scavenging; G, H and I – mechanism of ATP generation or acquisition. See text for details.

### Genome streamlining – amino acid auxotrophy

Genome streamlining refers to an adaptive reduction of genome size and complexity, possibly as a response to replication in a nutrient poor environment and/or to optimize the metabolic cost of biomass generation (Giovannoni et al., 2014; Bobay and Ochman, 2017). Despite uncertainty regarding underlying selective pressure(s), genome streamlining at the expense of metabolic capacity and plasticity is a hallmark of BOIPs (Andersson et al., 1998; Stephens et al., 1998; Seshadri et al., 2003; Brenner et al., 2021). From an energetic perspective, the relative benefit of genome streamlining to a pathogen can be appreciated based on the cost of biosynthesis of specific types of molecules. Intermediates of central carbon metabolism serve key roles as precursors for the biosynthesis of other molecules, including amino acids (Noor et al., 2010). Amino acids, nucleotides and fatty acids represent the most energetically costly molecules for any organism to synthesize. For *C. trachomatis*, tryptophan has been highlighted as the most energetically costly amino acid to produce (Carlson et al., 2006). Importantly, the cost of amino acid synthesis can be dependent on the carbon source used (Kaleta et al., 2013).

**Figure 3:**
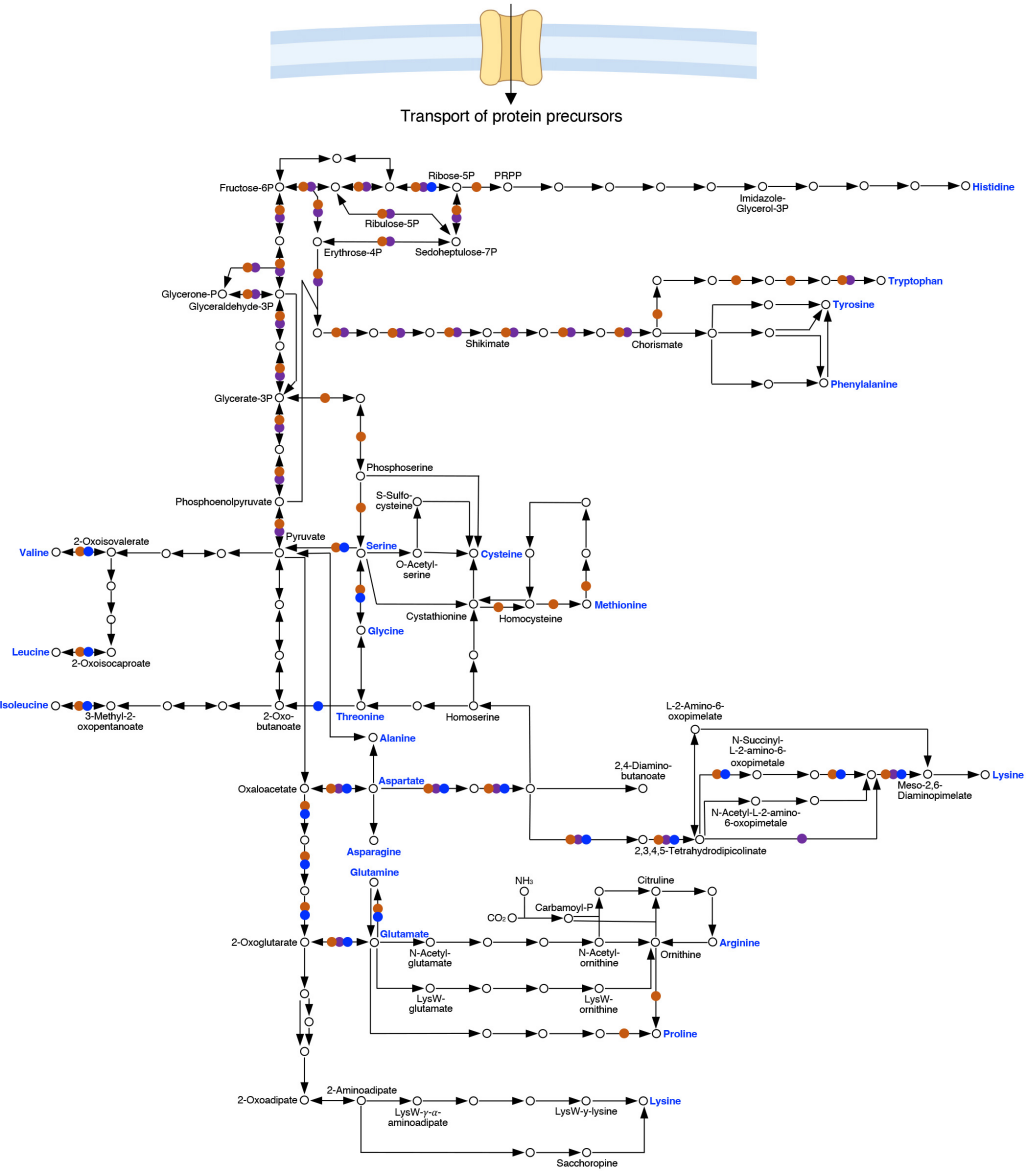
Comparison of pathways for biosynthesis of amino acids in *Coxiella*, *Chlamydia* and *Rickettsia*. Genome sequence analysis shows that the pathways for amino acid biosynthesis in *C. burnetii* (RSA493) (●), *C. trachomatis* (Bu/434) (●) and *R. prowazekii* (Madrid E) (●) are vastly different. Acquisition of protein precursors from the host cell, transporter specificities, and significance of such transport in virulence are largely unexplored areas of research for BOIPs.

In addition to the biosynthetic cost, the pathways and thus the number of enzymes required for biosynthesis can be extensive, which results in a significant increase in genome size for organisms that have retained more extensive biosynthetic capacity. Bioinformatic analysis of the capacity to synthesize amino acids illustrates key metabolic characteristics and biologically relevant differences between *C. burnetii*, *C. trachomatis*, and *R. prowazekii* (**Figure 3**) (Ogata et al., 1999). In general agreement with bioinformatic analysis, using a chemically defined medium not supplemented with specific amino acids, *C. burnetii* was demonstrated unable to grow beyond a 10-fold increase in cell numbers in media lacking any one of 11 amino acids (Sandoz et al., 2016). Comparative bioinformatic analysis of the capacity for biosynthesis of amino acids in *C. burnetii*, *C. trachomatis* and *R. prowazekii* shows a gradual decrease in biosynthetic capacity between these organisms. The absence of key central metabolic capacity in *Rickettsia*, including absence of glycolytic enzymes, underlies a major challenge for *de novo* biosynthesis in this genus.

As a phenotype, auxotrophy is not an absolute but rather entirely dependent on the availability of biosynthetic precursors and the ability of any organism to utilize such precursors. As an example, *C. burnetii* does not appear auxotrophic for either glutamate or glutamine in ACCM-D-based media (Sandoz et al., 2016), despite lack of glutamate synthase and thus a predicted inability to synthesize glutamate from glutamine. However, the expression of glutamate dehydrogenase (EC 1.4.1.2, reverse reaction) may fill the nutritional need for glutamate under conditions where the cell also produces α-ketoglutarate. Because protein synthesis largely takes place in the cytoplasm of eukaryotic cells, *Rickettsia* may experience less selective pressure to maintain pathways for amino acid synthesis than pathogens occupying replication vacuoles. Indeed, the pathways involved in amino acid synthesis reveals greater loss of biosynthetic capacity for *R. prowazekii* as compared to *C. burnetii* and *C. trachomatis* (**Figure 3**). Possibly as an adaptation to reduced capacity to synthesize protein precursors, BOIPs can show unique mechanisms to optimize utilization and recycling of amino acids. For example, in *C. trachomatis*, the Opp transporter has dual function as both an oligopeptide transporter serving the bacterium’s nutritional needs and at the same time functioning in peptidoglycan recycling (Singh et al., 2020).

### Interplay between physicochemical conditions, nutrient availability, and metabolic capacity

BOIPs inhabit niches that are known to differ in terms of physicochemical characteristics. For example, while the host cell cytoplasm has a neutral pH, the *C. burnetii* CCV luminal pH is acidic (Grieshaber et al., 2002; Samanta et al., 2019). Additional physicochemical variables relevant to the physiology of BOIPs include osmolarity, relative concentrations of ions associated with nutrient transport, as well as availability of oxygen (O_2_), carbon dioxide (CO_2_), and temperature. A branched respiratory chain that includes cytochrome *bd*, a terminal oxidase associated with reduced oxygen availability (Borisov et al., 2021), motivated analysis of *C. burnetii* oxygen requirements (Omsland et al., 2009). Even under nutrient conditions permissive to growth, *C. burnetii* does not replicate optimally unless O_2_ tension is reduced to microaerobic levels and CO_2_ is available (Esquerra et al., 2017). Like *C. burnetii*, requirements for growth of other BOIPs is likely to be as dependent on specific physicochemical conditions as critical nutrient availability.

As illustrated by the ability of *C. burnetii* to replicate under relatively simple nutrient conditions where amino acids and citrate (note: citrate is not required for growth) serve as the only macro-nutrients (Esquerra et al., 2017), *C. burnetii* may not fall into the category of a nutritionally fastidious bacterium. However, a requirement for specific nutrients and physicochemical conditions (i.e., pH, O_2_ and CO_2_) for optimal growth reflects adaptation to a unique replicative niche. It should also be noted that some genogroups of *C. burnetii* show poor cultivability under standard axenic culture conditions (Kersh et al., 2016), suggesting that isolates within the genus are metabolically diverse. Natural diversity in cultivability within the *Coxiella* genus could originate from extensive genome rearrangements (Beare et al., 2009) that affect gene expression or be a result of specific genome content. The high level of conservation between genomes of *C. trachomatis* serovars suggests this pathogen may show more uniform nutrient/cultivation requirements, as compared to *C. burnetii*. Serovar-specific ability to synthesize tryptophan *de novo* is a well-characterized metabolic difference between pathogenic *Chlamydia* species (Caldwell et al., 2003; Bommana et al., 2021). Loss of tryptophan synthase activity in ocular serovars of *C. trachomatis* is linked to loss of metabolic fitness due to accumulation of ammonia in indole-deficient environments (Sherchand and Aiyar, 2019).

*C. muridarum* has been shown to replicate in host cells maintained in the absence of oxygen, albeit at a slower rate than in host cells incubated under normoxic conditions (Sigar et al., 2020). A similar analysis showed that while *C. pneumoniae* replication is enhanced under microaerobic conditions, *C. trachomatis* is not affected (Juul et al., 2007). Analysis of *C. trachomatis* metabolic activity under axenic conditions showed enhanced activity over time under microaerobic conditions (2.5% O_2_) as compared to normoxic conditions (20% O_2_) for both the EB and RB cell forms (Omsland et al., 2012). It should be noted that *C. trachomatis*, *C. pneumoniae*, and *C. muridarum* all carry *cydAB*, which encodes cytochrome *bd*, a terminal oxidase typically used by bacteria under reduced oxygen availability (Borisov et al., 2021). Overall, these data point to clear effects of O_2_ tension on chlamydial metabolic activity and a positive effect of microaerobic oxygen availability. Similar to *C. burnetii* and *C. trachomatis*, cytochrome *bd* is also encoded by the *R. prowazekii* genome. In *E. coli*, expression of respiratory chain components is generally regulated by the FNR and ArcAB regulatory systems (Cotter et al., 1997; Levanon et al., 2005). The *C. burnetii* genome does not encode orthologs of these genes. It is possible that *C. burnetii*’s obligate intracellular lifestyle and adaptation to the specific conditions of the CCV has reduced the requirement for regulation and that *C. burnetii* responds by a more general upregulation of gene expression once within its replicative niche. Enhancement of *C. psittaci* axenic activity in the presence of CO_2_ (Weiss and Wilson, 1969) suggests that other species in the genus may also need CO_2_ for optimal activity and eventual axenic replication, as observed for *C. burnetii* (Esquerra et al., 2017).

*Coxiella* and *Chlamydia* are typically cultured at 37°C, generally consistent with their tissue tropisms in mammals. However, *Chlamydia* species show species and isolate-specific temperature preferences (Rota and Nichols, 1973; Janik et al., 2014). Additionally, under axenic conditions, *C. burnetii* also replicates efficiently at 27°C, albeit with an extended lag phase (Esquerra et al., 2017), potentially reflective of the organism’s ability to colonize ticks. Unlike *Coxiella* and *Chlamydia*, *Rickettsia* species are maintained between 28-37°C during infection of cultured cells, depending on the host cell type (e.g., mammalian vs arthropod) and pathogen species (Tello-Martin et al., 2018). For *R. prowazekii*, culture below 37°C does not necessarily correlate with optimal replication or the temperature of naturally infected tissue but rather a compromise between pathogen replication and maintenance of the infected host cells during infection (Pinkerton and Hass, 1932a, 1932b). Interestingly, temperature (25 vs 37°C) does not have a marked effect on gene expression in *R. rickettsii* (Ellison et al., 2009), although some effect of temperature has been noted for virulence-related genes (Galletti et al., 2016). Moreover, viability of *R. rickettsii* is better maintained at 25°C compared to 34°C during culture in Vero cells (Ellison et al., 2009), a finding potentially affected by host cell viability following infection at 25°C vs 34°C. A negative effect of elevated temperature on viability suggests that temperature could have a significant impact on the ability to culture *Rickettsia* species under axenic conditions. The effect of temperature on growth, virulence factor expression, and viability is likely regulated via a range of mechanisms including DNA and mRNA structure, thermo-sensitive changes in protein structure, transcription factors, and chaperone proteins (Lam et al., 2014).

### Micronutrient acquisition and metabolism

Micronutrients such as transition metals and vitamins are critical for normal metabolic functions in bacteria. For BOIPs, micronutrient acquisition is in part shaped by genome streamlining. Without biosynthetic pathways to synthesize NAD^+^ *de novo*, *C. trachomatis* has evolved substrate promiscuity in the ATP/ADP translocase Npt1_Ct_ to acquire NAD^+^ from the host (Fisher et al., 2013). Similarly, novel enzymes, including isoforms with less restrictive substrate specificity, have been identified as components of the pathway for tetrahydrofolate (an active form of vitamin B9) biosynthesis in *C. trachomatis* (Adams et al., 2014).

Iron is a critical micronutrient known to influence the expression of dozens of genes in free-living or facultative intracellular bacterial pathogens. Analysis in *C. burnetii* (Sanchez and Omsland, 2020) and *C. trachomatis* (Pokorzynski et al., 2019) have highlighted unique aspects or iron acquisition and metabolism in BOIPs. *C. trachomatis* utilizes the iron-dependent transcriptional regulator YtgR to integrate responses to iron starvation and tryptophan biosynthesis (Pokorzynski et al., 2019). In *C. burnetii*, apparent reliance on uptake of molecular iron via the Fe^2+^-specific FeoAB transporter has been linked to release of molecular iron from iron-containing molecules within the acidic microenvironment of the CCV (Sanchez and Omsland, 2020). *C. burnetii* cannot utilize heme as a source of iron but rather relies on *de novo* heme biosynthesis (Moses et al., 2017) in combination with acquisition of molecular iron (Moses et al., 2017; Sanchez and Omsland, 2020).

Responses to iron limitation also serve to illustrate a likely characteristic aspect of niche adaptation common to many BOIPs. While limiting iron access to *R. rickettsii* during infection of Vero cells using deferoxamine mesylate results in inhibition of growth, only 5 genes showed ≥ 3-fold differential expression in response to iron limitation (Ellison et al., 2009). Thus, *R. rickettsii* transcriptional responses to iron limitation appear similar to that observed in *C. trachomatis* whose response to iron limitation during (mid-cycle) intracellular growth following 6 h treatment with the chelator 2,2’-bipyridyl only involve 12 genes (Brinkworth et al., 2018). Complementary genome-wide transcriptional profiling to iron limitation is not available for *C. burnetii*, but combined bioinformatic and biochemical analysis of *C. burnetii* transcriptional responses to iron suggests the involvement of a limited number of genes in iron-induced stress responses (Briggs et al., 2008). As suggested for *R. rickettsii* (Ellison et al., 2009), BOIP responses to iron may reflect the parasites’ residence in relatively stable intracellular niches with limited requirements to respond to physiologically significant changes in iron availability. This stands in stark contrast to BOIP responses to other nutritional cues, including IFNγ-induced tryptophan starvation in *C. pneumoniae* where transcriptional activity is globally upregulated (Ouellette et al., 2006).

Analysis of *C. burnetii* growth in chemically defined media combined with metabolic pathway reconstruction suggest *C. burnetii* can synthesize most vitamins (Seshadri et al., 2003; Esquerra et al., 2017). Interestingly, *C. burnetii* has two orthologous *bioC* (Moses et al., 2017), allowing the initial step in biotin (vitamin B7) production. Because chemical inhibition of biotin synthesis inhibits *C. burnetii* replication, *de novo* biotin biosynthesis is likely critical in *C. burnetii*. Bacteria in the *Rickettsia* genus are unable to synthesize several cofactors and B vitamins *de novo* (Driscoll et al., 2017), whereas members of the *Chlamydia* genus are predicted to exhibit species-specific capacity for biotin synthesis (Voigt et al., 2012). Although *C. trachomatis* and *C. psittaci* can synthesize folates, strains have different capacities to scavenge folates from the host cell (Fan et al., 1992). As postulated for *Rickettsia* (Driscoll et al., 2017), deficiencies in cofactor and vitamin biosynthesis likely contribute to the parasitic nature of BOIPs.

## BOIP-host interactions

### Nutritional virulence and nutrient acquisition

BOIPs show remarkable diversity regarding their host cell interactions. Some aspects of these interactions are shaped by the interplay between pathogen metabolic capacity and continued pathoadaptation of BOIPs to their intracellular niches (Fuchs et al., 2012; Eisenreich et al., 2017). The concept of nutritional virulence (Kwaik and Bumann, 2013) frames nutrient acquisition by intracellular pathogens in terms of pathogen virulence mechanisms. For BOIPs, strategies for nutrient acquisition are interconnected with niche adaptation and genome streamlining. Advances in genetic manipulation of some BOIPs has allowed for the identification of specific genes with roles in nutrient acquisition. The molecular mechanisms for how *Coxiella* (Voth and Heinzen, 2007; Schaik et al., 2013; Kohler and Roy, 2015; Larson et al., 2016), *Chlamydia* (Elwell et al., 2016; Fischer and Rudel, 2018; Rother et al., 2019; Triboulet and Subtil, 2019), and *Rickettsia* (Driscoll et al., 2017; McGinn and Lamason, 2021; Voss and Rahman, 2021) interact with their respective host cells during infection has been discussed elsewhere. While this review primarily focuses on nutrient utilization and responses to nutrient limitation, recent findings regarding strategies for nutrient acquisition by BOIPs during infection warrant some discussion.

Nutritional virulence encompasses bacterial virulence strategies that target host processes to enhance pathogen access to nutrients. Because factors other than access to nutrients impact replication of BOIPs, especially those that reside in replication vacuoles, it can be challenging to distinguish the significance of underlying mechanisms. For example, in *C. burnetii*, several effectors are critical for normal CCV biogenesis and thus intracellular replication (Larson et al., 2013; Newton et al., 2014; Crabill et al., 2018) without necessarily affecting pathogen access to nutrients. Also, some genes, including CBU2028, are specifically linked to CCV biogenesis and not pathogen intracellular replication (Crabill et al., 2018), demonstrating that CCV size and pathogen intracellular replication are not necessarily linked. Recruitment of autophagic vesicles to the CCV may have more significance for recruitment of membranes to aid CCV expansion than pathogen replication, despite some evidence pointing to a direct effect on *C. burnetii* replication (Pareja et al., 2017). This may also hold true for *C. trachomatis*, which grows equally well in autophagy competent or incompetent mouse embryonic fibroblasts suggesting autophagy is not a critical host process for chlamydial growth (Ouellette et al., 2011). Comparison between *C. trachomatis* and *C. pneumoniae* has shown that these two species differ in their strategies to obtain protein precursors from the host cells. *C. pneumoniae* is primarily reliant on lysosome-derived peptides while *C. trachomatis* shows a preference for cytosolic amino acids (Ouellette et al., 2011). Moreover, the mechanisms by which *Chlamydia* obtains protein precursors from host cells can change throughout the developmental cycle. Chlamydial downregulation of host p53, a negative regulator of the PPP enzyme glucose-6P dehydrogenase, serves to enhance PPP activity in *Chlamydia*-infected host cells (Siegl et al., 2014). The resulting pathogen-dependent stimulation of host metabolism may promote a nutritionally favorable environment for the pathogen (Rother et al., 2019). Infection of mouse oviduct epithelial cells by *C. muridarum* results in upregulation of hexokinase II (George et al., 2016), consistent with pathogen-dependent stimulation of (host) production of both glucose-6P and ATP, the latter a result of glucose-6P oxidation.

The unfolded protein response (UPR) is a reaction to endoplasmic reticulum (ER) associated stress in eukaryotic cells (Celli and Tsolis, 2014). Some pathogens exploit the UPR and downstream ER-associated degradation (ERAD) of unfolded proteins as a source of amino acids. In *C. burnetii*-infected THP-1 macrophages, inhibition of ER stress by tauroursodeoxycholic acid reduces expansion of the CCV but does not affect pathogen replication (Brann et al., 2020), suggesting the UPR and ERAD are not critical for *C. burnetii* to access protein precursors. Nevertheless, normal CCV expansion and *C. burnetii* replication depend on activity of the UPR-related translation initiation factor eIF2α, the phosphorylation of which is reduced upon infection with bacteria unable to secrete type IVB secretion system effectors (Brann et al., 2020). Because phosphorylation of eIF2α enhances autophagy (Kouroku et al., 2006; Humeau et al., 2020), *C. burnetii* manipulation of UPR-related signaling may enhance pathogen recruitment of autophagosomes to the CCV and aid replication.

*C. burnetii* is critically dependent on moderately acidic pH for optimal transport and metabolism of specific metabolites (Hackstadt and Williams, 1981a) and replication (Esquerra et al., 2017). The discovery that *C. burnetii* actively manipulates the host cell to maintain CCV luminal pH to prevent activity of hydrolytic enzymes (Samanta et al., 2019) suggests *C. burnetii* is essentially replicating at a pH during intracellular replication that is sub-optimal for pathogen metabolism. The overall high metabolic plasticity of *C. burnetii*, illustrated by the ability to replicate in axenic medium composed largely of amino acids (Esquerra et al., 2017), may be important for *C. burnetii* to balance metabolic fitness within a CCV that is manipulated to maintain a sub-optimally high pH for pathogen metabolic activity.

Building on the discovery that EBs and RBs primarily utilize different mechanisms to obtain energy for protein synthesis (i.e., scavenging of host-derived ATP by the RB versus oxidation of glucose-6P by the EB), Grieshaber and colleagues showed that the chlamydial EB can use chemically diverse molecules, including amino acids and ATP, to maintain infectivity (Grieshaber et al., 2018). Although EBs might prefer glucose-6P to support protein synthesis (Omsland et al., 2012), the ability of EBs to respond to ATP suggests *Chlamydia*-dependent release of ATP from host cells (Pettengill et al., 2012; Yang et al., 2021) is a mechanism to enhance pathogen infectivity. As such, release of ATP by infected cells could have an impact on dissemination and disease progression.

Mechanisms of nutritional virulence likely differ between pathogens that replicate in the host cell cytosol and those that establish replication vacuoles (e.g., transporter requirements). Comparative analyses aimed at understanding differences in strategies employed by BOIPs for nutrient acquisition within specific intracellular niches will be important to understand mechanisms of pathoadaptation among these unique bacterial pathogens.

### Metabolic capacity as a virulence determinant of amphotropism

The virulence of bacterial pathogens is conferred by a combination of essential and subtle virulence determinants. For example, in *Coxiella*, secretion of effector molecules via a type IVB secretion system (Chen et al., 2010; Carey et al., 2011; Beare et al., 2012; Larson et al., 2016) is critical for establishment of the pathogen’s replication vacuole. That metabolic capacity can also affect pathogen virulence is well established; however, identifying the specific mechanism(s) for how a metabolic defect affects virulence can be challenging.

All BOIPs are associated with highly specific replication niches. The large diversity in apparent metabolic capacity (as deduced from genome sequence analysis) among BOIPs suggest that the various niches occupied by BOIPs represent vastly different nutritional environments. BOIPs of the genera *Coxiella*, *Chlamydia*, and *Rickettsia* are all to some degree amphotropic—capable of infecting different host organisms and host cell types—in nature. For example, *C. burnetii* has been shown to naturally colonize a wide range of animals [e.g., birds, cows, goats, and ticks (Canevari et al., 2018; Tokarevich et al., 2018; Tomaiuolo et al., 2020; Turcotte et al., 2021)] in addition to various tissues within infected animals (Roest et al., 2012; Gregory et al., 2019). Direct comparative analysis of the ability of different serovars of *C. trachomatis* to infect different host cell types indicates that the invasive *C. trachomatis* serovar L2, causing lymphogranuloma venereum, is moderately amphotropic compared to serovars A and D (Faris et al., 2019).

Transposon mutant libraries of *C. burnetii* reveal genes encoding metabolic functions whose disruption affect infection and/or intracellular replication (Martinez et al., 2014; Newton et al., 2014). Several genes have functions related to processes discussed in this review, including CO_2_ metabolism (carbonic anhydrase, CBU0139), respiration (cytochrome c oxidase, CBU1038-1040), glycolysis (glucose-6P isomerase, CBU0848), and metabolism of phosphorylated glucose (UTP-glucose-1-phosphate uridylyltransferase, CBU0849). Moreover, metabolic plasticity conferred by *pckA* has revealed specific virulence defects during *C. burnetii* intracellular replication in certain host cell types (Sanchez et al., 2021).

*C. burnetii* shows isolate-specific plasmid carriage (Long et al., 2019). Luo and colleagues recently identified a role for the QpH1 plasmid of *C. burnetii* during colonization of murine bone marrow-derived macrophages (Luo et al., 2021). It is not clear whether the role of the QpH1 plasmid in *C. burnetii* host colonization relates to virulence factor secretion or pathogen metabolic functions. Nevertheless, diversity in plasmid carriage among *C. burnetii* isolates may influence *C. burnetii* amphotropism based on potential significance of specific plasmid-associated genes in *C. burnetii* metabolic activities. Because genome architecture can influence gene expression, extensive differences in genome organization, including sequences containing metabolic genes, among *C. burnetii* isolates (Beare et al., 2009) combined with isolate-specific disease characteristics is consistent with a role of genome architecture in pathogen metabolism. We expect that research over the next decade will continue to reveal greater significance of metabolic functions in BOIP virulence and begin to provide mechanistic information related to a range of metabolic virulence determinants.

### Use of physiological (host cell) media in the analysis of BOIP-host interactions

Although use of host cell-free culture is an invaluable tool to physiologically separate BOIPs from their host cells, ultimately, the physiology of BOIPs is intertwined with that of the host cell. As such, analysis of metabolic and physiological characteristics of BOIPs will require verification of biological relevance using (*in vivo*) animal or (*ex vivo*) cell culture models.

For any *ex vivo* analysis of BOIPs, the nutrient composition of cell culture media may affect, and in some cases compromise, the utility of cell culture models to resolve phenotypes of mutants. For example, medium nutrient composition can result in accidental chemical rescue of a genetic defect. To explore how the cell culture medium can impact pathogen fitness during infection of different host cell types, a “physiological medium” (Cantor, 2019) based on the nutrient composition of interstitial fluid was designed for use with *C. burnetii* as the model BOIP (Sanchez et al., 2021). Use of Interstitial Fluid-modeled Medium, IFmM, proved to affect *C. burnetii* fitness in some but not all cell culture models. Pathogen metabolic capacity, as assessed by comparing strains capable of undergoing gluconeogenesis or not, also showed host cell type-dependent differences (Sanchez et al., 2021). Overall, this underscores the significance of the interplay between pathogen metabolic capacity and the nutritional context of the host cell.

In the study of *Chlamydia* species, the protein synthesis inhibitor cycloheximide is often used to promote pathogen replication by suppressing host cell protein synthesis (Alexander, 1968), overall enhancing bacterial replication by reducing host cell competition for nutrients. Notably, cycloheximide results in (host) carbon source-dependent effects on the generation of *C. trachomatis* EBs (Iliffe-Lee and McClarty, 2000). Because use of cycloheximide promotes chlamydial fitness during interaction with a host cell, analysis of chlamydial physiology, especially with strains that have metabolic defects, may be best done using cell culture models maintained in the absence of cycloheximide.

### Sensing the (state of the) host

Any microbial pathogen must be able to respond to cues from the host to optimize its virulence potential. This may be most obvious in the context of responding to nutrient availability and physicochemical conditions such as pH and temperature. As suggested for *C. burnetii* (Esquerra et al., 2017), the integration of several distinct physicochemical and nutritional signals could serve as a mechanism for this organism to identify a suitable replicative niche. Similarly, *Chlamydia* and *Rickettsia* species may sense availability of ATP, a molecule largely limited to metabolically active cells, as a signal that environments suitable for growth have been encountered.

Less obvious, although the phenomenon has been known for decades (Morse and Fitzgerald, 1974; Hughes and Sperandio, 2008), is the ability of bacteria to sense and respond to mammalian (steroid) hormones, molecules unique to the host organism they infect. Unlike molecular nutrients such as glucose and amino acids that are maintained at relatively steady levels within eukaryotic host cells, hormone levels typically fluctuate, thus reflecting altered physiological states of the host organism. *C. burnetii*, a bacterium known to colonize placental tissue, has been shown to establish infection in cultured placental trophoblasts (Amara et al., 2010; Howard and Omsland, 2020), a major source of the steroid hormone progesterone during pregnancy. In ovariectomized mice, *C. burnetii* shows elevated loads in spleen and liver tissue (Leone et al., 2004), suggesting *C. burnetii* responses to mammalian steroid hormones has clinical significance. Data from host cell-free analysis of *C. burnetii* responses to progesterone suggest that this hormone has a direct inhibitory effect on *C. burnetii* activity (Howard and Omsland, 2020). However, in the context of animal infection and considering hormonal effects on the immune system (Bereshchenko et al., 2018; Patt et al., 2018), it is likely that steroid hormones also have indirect effects on pathogen activity and pathogenicity. Thus, while progesterone may directly inhibit *C. burnetii* activity, hormonal effects on host immunity may significantly confound how bacteria respond to mammalian hormones. Similar to *C. burnetii*, *C. abortus* shows tropism for placental tissue (Essig and Longbottom, 2015) and may also respond to host-derived hormones or be affected by hormone-associated signaling during colonization of placental tissue.

## BOIP physiology

### Cell forms and developmental transitions

**Figure 4:**
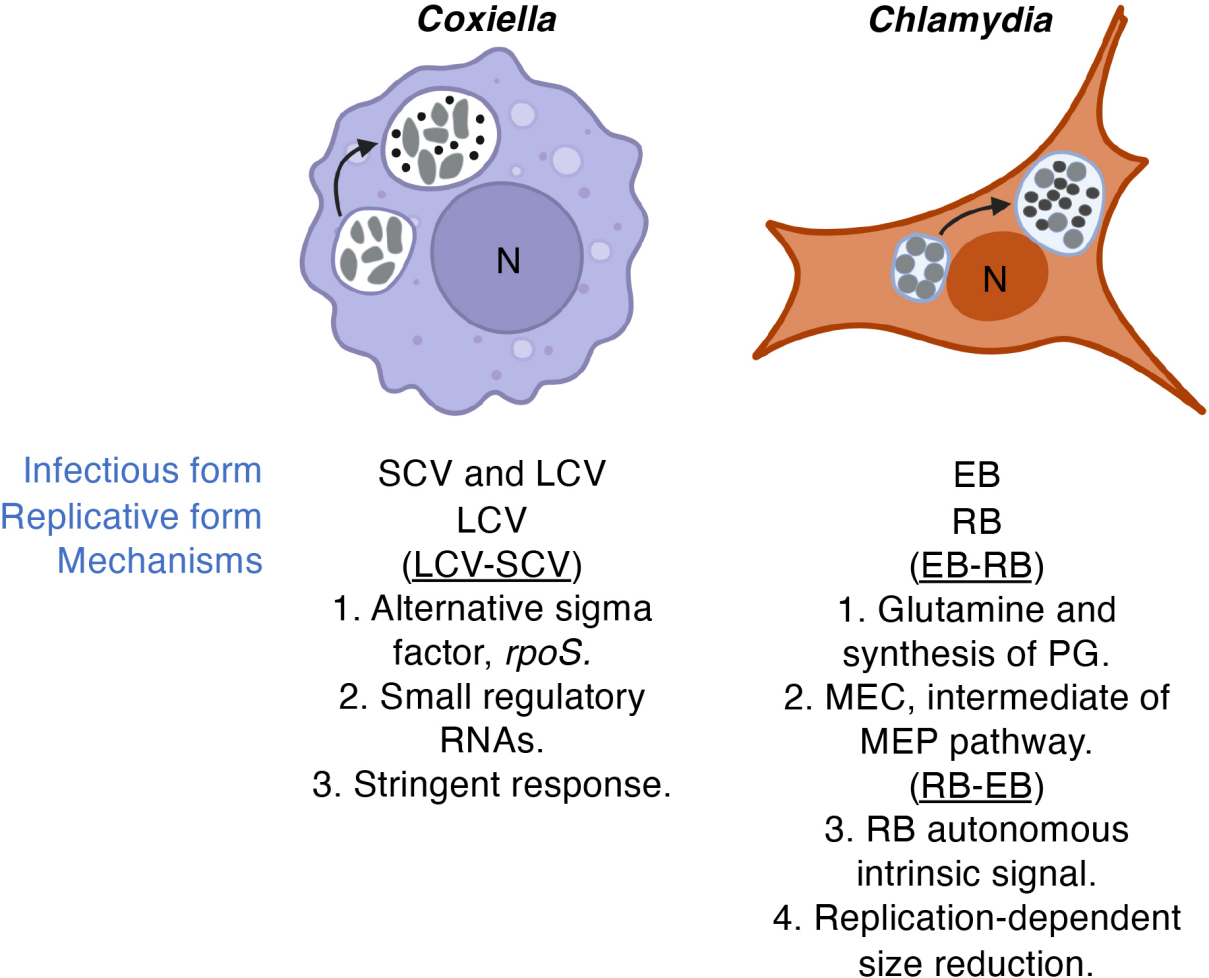
Developmental transitions. While recent advances based on molecular genetics are starting to shed light on the mechanism(s) of developmental transitions in *Coxiella* and *Chlamydia*, the genes and regulatory networks involved remain elusive. Leading ideas for mechanisms underlying developmental transitions are highlighted. LCVs and RBs vs SCVs and EBs are indicated by larger gray cells or smaller dark calls, respectively. Infectious form relates to infectivity of cultured cells. The direction of the developmental cycle is indicated in parentheses. "PG" and "MEC" denote peptidoglycan and 2-*C*-methylerythritol 2,4-cyclodiphosphate, respectively.

The physiology of BOIPs is interconnected with their metabolic capacity and plasticity. Some BOIPs, including *Coxiella*, *Anaplasma*, *Ehrlichia* and *Chlamydia*, undergo morphological transitions during their life cycles, further complicating the intricate relationships they maintain with their respective host cells. The developmental cycles of *C. burnetii* and *C. trachomatis* with leading ideas for underlying mechanisms are illustrated in **Figure 4**. Coleman and colleagues (Coleman et al., 2004) connected the LCV form of *C. burnetii* to this pathogen’s replicative phase while the SCV was shown to be a non-replicative stationary phase form. Current understanding of the physiological role of the *C. burnetii* SCV is limited but certain spore-like characteristics are consistent with environmental stability. In cell culture, both the *C. burnetii* SCV and LCV forms are infectious.

The physiological basis for developmental transitions in *Chlamydia* species appear distinct from those of *C. burnetii*. For example, because only the chlamydial EB form is infectious, re-generation of the chlamydial EB is critical for new rounds of infection. Despite lack of cell division by the chlamydial EB, in *C. trachomatis*, the EB continues to synthesize protein during infection of cultured cells (Grieshaber et al., 2018), consistent with a requirement for continued “maintenance metabolism” after RB-EB differentiation is completed.

The question of what triggers transition of the replicative LCV and RB forms of *C. burnetii* and *C. trachomatis* to their respective non-replicative SCV and EB forms remains largely unanswered. That nutrient availability can control morphological transitions in BOIPs is supported by the mechanism of developmental transitions in the intracellular bacterium *Legionella pneumophila*, a phylogenetic relative of *C. burnetii*. In *L. pneumophila*, the gene *phtA*, encoding a major facilitator superfamily transporter, influences developmental transitions in response to threonine availability (Sauer et al., 2005; Fonseca and Swanson, 2014). In part relying on axenic techniques, Rajeve et al. have produced promising data regarding a potential trigger for EB-RB development in *C. trachomatis* (Rajeeve et al., 2020). This group reported an interconnection between metabolism of glutamine and synthesis of peptidoglycan required for cell division in *C. trachomatis* (Liechti et al., 2016), establishing a critical role for glutamine in EB germination. Moreover, 2-*C*-methylerythritol 2,4-cyclodiphosphate (MEC), an intermediary metabolite of the methylerythritol phosphate (MEP) pathway of isoprenoid synthesis, stimulates dissociation of the chlamydial histone-like protein Hc1 from DNA thus promoting chromatin decondensation (Grieshaber et al., 2004). Because the initial step of the MEP pathway involves pyruvate and glyceraldehyde 3-phosphate (G3P), both intermediates of the central metabolic machinery, activation of central metabolism to generate pyruvate and G3P could serve as a metabolic trigger for decondensation of the EB nucleoid, a critical step in EB-RB development. To further elucidate the driving force behind *C. trachomatis* RB-EB transitions, Chiarelli et al. tested if signals extrinsic (i.e., extracellular) or intrinsic (i.e., intra-bacterial) to *C. trachomatis* are likely to be responsible for triggering RB-EB developmental transitions using mathematical models. Interestingly, they identified the triggering signal(s) to be a cell-autonomous intrinsic signal and thus not likely to be an environmental signal such as a nutrient (Chiarelli et al., 2020). Lee and colleagues established that replication-dependent size reductions of the RB controls the timing of RB-EB conversion, also in the absence of an external signal (Lee et al., 2018). It is possible that signals and mechanisms governing EB-RB versus RB-EB transitions are different in nature.

Numerous genes were implicated in developmental transitions of *C. trachomatis* in experiments conducted on temperature sensitive mutants (Brothwell et al., 2016). The influence of several genes in chlamydial morphological differentiation suggests redundancy in regulation. Similarly, potential involvement of small RNAs (Warrier et al., 2014) and the recently discovered significance of the alternative sigma factor RpoS in *C. burnetii* developmental transitions (Moormeier et al., 2019) also suggest the involvement of a larger number of genes and regulatory redundancy in the process. Regardless of what the nature of the trigger(s) for developmental transitions in (certain) BOIPs may be, understanding the makeup of the response network that allows for the orchestrated change in gene expression required to transform one cell form into another is a focus of current research.

### Mechanisms for sensing and responding to nutrient availability

The stringent response (SR) was identified as a regulatory mechanism that interconnects amino acid availability with RNA synthesis by providing “stringent” control of RNA synthesis under conditions of amino acid limitation (Cashel, 1969; Cashel and Gallant, 1969). The signaling nucleotide (p)ppGpp, synthesized by proteins of the RelA/SpoT homolog (RSH) family (Hauryliuk et al., 2015), and a range of co-regulatory proteins (Steinchen et al., 2020), make up the SR. In *L. pneumophila*, the response network involved with signaling amino acid availability via PhtA may be the SR (Sauer et al., 2005). Both *phtA* (Sauer et al., 2005) and an apparently intact SR network is found in *C. burnetii*, suggesting amino acid availability may also be directly involved in developmental transitions in this organism.

Analysis of the *C. trachomatis* genome suggested absence of a SR based on lack of enzymes for synthesis of (p)ppGpp (Ouellette et al., 2006). While genes for ppGpp synthesis are absent, chlamydial genomes do encode DksA, a SR-related transcriptional regulator known to work in concert with (p)ppGpp (Paul et al., 2004; Magnusson et al., 2007; Ross et al., 2016). A gene encoding GreA, a protein that in *E. coli* can functionally substitute for DksA (Vinella et al., 2012), has also been annotated in *C. trachomatis*, but the gene is larger than the *E. coli* ortholog and sequence homology is minimal. Overexpression of DksA in *C. trachomatis* has been shown to reduce generation of infectious EBs (Mandel et al., 2021). Curiously, the chlamydial *dksA* ortholog does not functionally complement the growth defect observed in *E. coli* during culture in a nutritionally minimal medium (Mandel et al., 2021), suggesting remnants of the SR machinery in *C. trachomatis* have evolved to acquire unique functions in this pathogen. Transcriptional analysis of *Chlamydia* under amino acid starvation has also revealed an apparent uncoupling of amino acid availability and transcriptional activity (Ouellette et al., 2006), again consistent with absence of “stringent” regulation of RNA synthesis. Ouellette and colleagues built on these findings and investigated the idea that chlamydial transcription during IFNγ-induced tryptophan limitation could be controlled via the density of tryptophan codons (Ouellette et al., 2016). Though it is unclear how codon content for an amino acid would regulate transcription, especially when the density of tryptophan codons does not absolutely correlate with transcript levels, the regulatory scheme points to chlamydial adaptation to amino acid starvation in the absence of a SR.

Direct comparison of transcriptional profiles of *C. trachomatis* cultivated in the presence or absence of IFNγ identified numerous differentially expressed genes (Belland et al., 2003a), consistent with chlamydial adaptation to an environment where an orchestrated response to tryptophan limitation is necessary for optimal fitness. In addition to the impact host-dependent regulation (i.e., synthesis vs IFNγ-dependent breakdown) of tryptophan can have on *C. trachomatis*, the host microbiota may also affect the *Chlamydia*-host interaction. Specifically, indole-producing species of the genus *Prevotella*, associated with bacterial vaginosis, can promote replication of *C. trachomatis*, thus potentially counteracting the effect of IFNγ-dependent control of tryptophan availability (Ziklo et al., 2016). Like *C. trachomatis*, intracellular replication of *C. burnetii* is also restricted by treatment of host cells with IFNγ via indolamine 2,3-dioxygenase 1-mediated breakdown of tryptophan (Ganesan and Roy, 2019). Interestingly, the inhibitory effect of IFNγ on the growth of *R. prowazekii* in human fibroblasts could not be rescued by addition of tryptophan to the growth medium, suggesting the involvement of alternative IFNγ-mediated inhibitory mechanisms (Turco and Winkler, 1986).

In the (facultative) intracellular pathogen *L. pneumophila*, the functional significance of (p)ppGpp and DksA have been demonstrated for pathogen differentiation between cell forms (Dalebroux et al., 2010). Given both phylogenetic and physiological relatedness, including morphological transitions, *C. burnetii* is also likely to rely on a functional SR machinery for normal physiological function. Although reliance on a SR by bacteria of the *Rickettsia* genus for the purpose of responding to amino acid availability has not been tested experimentally, genetic complementation of a truncated *relA*/*spoT* gene in a strain of *R. rickettsii* Iowa that produces lytic/clear plaques, restored the non-lytic/opaque phenotype of the plaques (Clark et al., 2011). This demonstrates the effect of *relA*/*spoT* expression in *R. rickettsii* Iowa and indicates the function of a response network that includes (p)ppGpp in *Rickettsia*. Interestingly, given the effect of (p)ppGpp levels on gene expression in free-living model organisms including *E. coli* (Sanchez-Vazquez et al., 2019), *relA*/*spoT* competence did not affect gene expression in *R. rickettsii* Iowa (late log phase) (Clark et al., 2011). The gene(s) annotated as *relA*/*spoT* in *Rickettsia* are shorter than the typical *relA* and *spoT* genes described in other organisms but could still encode functional (p)ppGpp synthesizing proteins because the synthase domain only accounts for a smaller part of the proteins (Andersson et al., 1998; McLeod et al., 2004; Clark et al., 2011).

The functional significance of the SR goes far beyond responses to amino acid limitation (Boutte and Crosson, 2013), the stressor first identified as a trigger for this response network (Borek, 1956; Cashel, 1969). Thus, molecular remnants of the SR in BOIPs, including *Chlamydia* that appear to encode a severely reduced system, may be responsive to stressors other than amino acids. Importantly, the retention of SR-related genes including *dksA* and *greA* in BOIPs could be driven by a role for these genes in processes other than those associated with the SR. For example, deletion of *dksA* increases accumulation of drug-induced double-stranded DNA breaks (Sivaramakrishnan et al., 2017), and both DksA and GreA/B can help resolve replication and transcription conflicts (Tehranchi et al., 2010; Satory et al., 2015). GreA has additionally been shown to serve as a chaperone (Li et al., 2012). Related yet unique functions of DksA and GreA/B is consistent with diverse selective pressures to retain a combination of SR-related genes in BOIPs. Suppression of the growth defect produced by (p)ppGpp deficiency by overexpression of *dksA* and *greA* (Vinella et al., 2012) illustrate their potential functions in the absence of a (p)ppGpp-driven SR. A comparison of SR-related genes in *Coxiella*, *Chlamydia* and *Rickettsia* is shown in **Table 2**. Because the genomes of these organisms all contain at least some components of what may be described as the canonical machinery consisting of *relA*, *spoT*, *dksA* and *greA*, unique adaptation of specific components of the network appears likely. As indicated, the predicted GreA protein of *C. trachomatis* is unusually large (CTL0004; 715 amino acids) compared to that of *E. coli* (b3181; 158 amino acids), suggesting the *C. trachomatis* protein is not functionally comparable to the *E. coli* protein.

**Table 2:** SR and related genes in *Coxiella*, *Chlamydia* and *Rickettsia*.

Gene ID	*Ec*	*Cb*	*Ct*	*Rp* ^1^
**SR proper** *relA* *spoT* *dksA* *greA* *greB* **SR related** *obgE/cgtA* ^3^ *dnaK* ^4^ *nirD* ^5^ *gppA* ^6^	+ + + + + + + + +	+ + + + – + + – –	– – + +^2^ – + + – +/–^7^	– +^1^ + + – + + – +

*Coxiella burnetii* (*Cb*), *Chlamydia trachomatis* (*Ct*), *Rickettsia prowazekii* (*Rp*). Information for the free-living model organism *E. coli* (*Ec*) is included for context.

^1^ *R. prowazekii* Madrid E appears to have one functional and one non-functional copy of (bifunctional) *spoT*. Some *Rickettsia* species are annotated to have several copies of spoT. A similar complement of genes is found in the spotted fever group *R. rickettsii* (NC009882.1).

^2^ *greA* in *C. trachomatis* is unusually large compared to orthologs in other bacteria.

^3^ObgE/CgtA affects the ratio of (p)ppGpp and ppGpp (Persky et al., 2009).

^4^ *dksA* can suppress the phenotype of *dnaK* deletion (Kang and Craig, 1990).

^5^NirD can bind to and inhibit the activity of RelA (Léger et al., 2021).

^6^GppA hydrolyses the pentaphosphate (p)ppGpp to ppGpp (Somerville and Ahmed, 1979; Keasling et al., 1993).

^7^ *gppA*/GppA is annotated in select isolates of *C. trachomatis* (e.g., CRH63140.1).

Analysis of *greA*/*greB*/*dksA* triple mutants in a ppGpp^0^ strain of *E. coli* showed recovery of growth on minimal medium (not containing amino acids) upon complementation with either *greA* or *dksA* (Vinella et al., 2012). The redundancy demonstrated for the SR in *E. coli*, as this relates to response to amino acid starvation, hints at the molecular composition of a minimal “SR” in BOIPs, or other bacteria with reduced genomes. In addition to prototypical response systems for nutrient sensing, bacteria with a streamlined genome may well utilize expression of e.g., transporters as an “indirect” way of regulating physiological responses to nutrient availability.

## Opportunities for discovery

**Figure 5:**
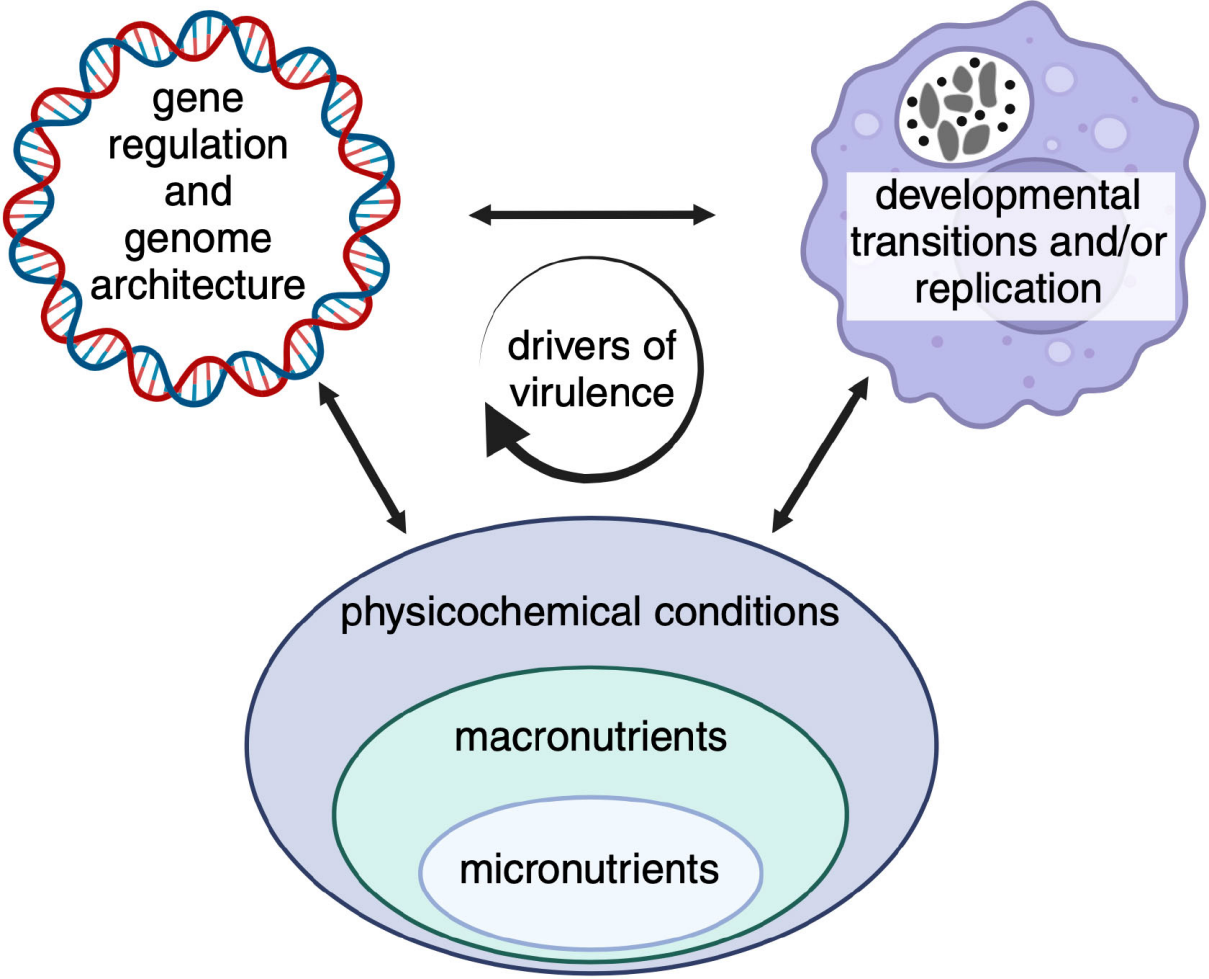
Opportunities for discovery. Certain areas of research appear especially relevant given recent technical and/or scientific advances. While these areas may be scientifically distinct, regulatory mechanisms are biologically intertwined and expected to influence pathogen virulence characteristics. Indicated micronutrients include, trace metals, vitamins and co-factors, macronutrients carbon sources and amino acids, and physicochemical conditions pH, oxygen and carbon dioxide.

Over the past decade, analysis of molecular mechanisms of *C. burnetii* biology has been transformed with the combined access to a full complement of tools for genetic manipulation and axenic culture, allowing isolation of mutants with defects in genes needed for host cell infection and/or intracellular replication. Given recent advances in similar research tools for several other BOIPs, the next decade is likely to change the study of BOIPs. Some areas of research may be especially important and scientifically fruitful. **Figure 5** illustrates the interrelatedness between the research areas described below.

### Analysis of (metabolic) interactions between BOIPs and their host cells

Development of more sensitive techniques (e.g., RNA-Seq) for analysis of transcriptional responses have resulted in several studies of BOIP gene expression during host interactions. Regarding metabolic responses, metabolomic analyses of BOIPs have revealed patterns and complexities in nutrient utilization (Häuslein et al., 2017). An underexplored area and natural next step in understanding the physiology of BOIPs is combined omics analyses aimed at correlating gene and/or protein expression with the flux of metabolic intermediates during the pathogens’ life cycle. This is especially relevant for organisms that undergo developmental transitions between cells forms as data based on both gene expression and metabolic activities can identify signaling networks involved in regulating such transitions. *C. burnetii* would be a great model for analysis given the transition of this organism between cell forms under axenic conditions (Sandoz et al., 2014; Esquerra et al., 2017), likely also required to obtain sufficient material for optimal analysis.

Metabolic interactions between BOIPs and host cells also extends to pathogen-dependent manipulation of host transcription factors that regulate host cell metabolism. For example, *C. burnetii* affects the stability of the transcription factor HIF1α as well as HIF1α-regulated target genes involved in metabolism (Hayek et al., 2022). A recent analysis of host cell chromatin structure during infection with *C. trachomatis* (Hayward et al., 2020) provides a general view of how this pathogen impacts the host response to infection, including at the metabolic level. As discussed (Czyż et al., 2014; Rother et al., 2019), a thorough understanding of how and why BOIPs depend on a host cell for replication can be exploited to design host-directed antibacterial therapies.

### Genome structure, physiology and virulence

Genome sequencing efforts have produced critical information about the genomes of several species and/or isolates of BOIPs. Though BOIPs exemplify a fascinating range of apparent metabolic capacity and genome stability, how these features affect pathogen physiology is largely unexplored. For example, how do differences in genome architecture between *C. burnetii* isolates (Beare et al., 2009) affect metabolic capabilities and how might such differences influence metabolic fitness, amphotropism, and virulence? In *Chlamydia*, genome sequence analysis show that metabolic capacity can be both species and isolate/strain specific (Voigt et al., 2012). Analysis of virulence following genetic complementation of metabolic capacity, as illustrated by ectopic expression of glucose-6P dehydrogenase (Sanchez and Omsland, 2021) and catalase (Mertens and Samuel, 2012) in *C. burnetii*, is one way to assess the selective pressures driving genome streamlining in BOIPs. Understanding how metabolic capacity correlates with niche adaptation, restriction and host range seems attractive avenues for basic research.

### Nutrient availability, replication, and morphological transitions

The list of potential genes, regulatory networks and mechanisms by which BOIPs may sense and respond to nutrient availability is expansive. The requirement for moderately acidic pH in *Coxiella* nutrient transport and metabolic activation (Hackstadt and Williams, 1981a), and ATP scavenging in *Chlamydia* are among the few mechanisms that have been experimentally tested and independently verified for how BOIPs regulate their metabolism. 

*Coxiella*, *Chlamydia* and *Rickettsia* exhibit vastly different replication rates, a physiological characteristic implicated in virulence. As illustrated by analysis of the slow growing pathogen *Mycobacterium tuberculosis* (Beste et al., 2009), numerous genes can influence replication rate in bacteria. Capacity to synthesize protein and thus ribosome content has been implicated in this process. In *R. prowazekii*, despite only having single copies of genes encoding 16S or 23S rRNA, ribosome content has been described as an unlikely bottleneck in *R. prowazekii* replication (Winkler, 1995). The biological basis for replication rate and potential significance in virulence should be directly testable via currently available research tools for at least some BOIPs.

Several BOIPs undergo developmental transitions between cell forms. In part driven by the ability to genetically manipulate the organisms, both *Coxiella* and *Chlamydia* are emerging as attractive models to study the genetic and biochemical basis for such transitions. While regulation of developmental transitions in *C. burnetii* is likely to align with findings in *L. pneumophila*, including a role for the SR (Dalebroux et al., 2010), lack of a SR in *C. trachomatis* (Ouellette et al., 2006) and uncertainty regarding how this organism integrates responses to nutrient availability with critical physiological processes (e.g., developmental transitions) offer opportunity for significant discovery.

### Micronutrients

Despite obvious physiological significance, the effect and metabolism of micronutrients is an understudied aspect of BOIP biology. Technical challenges in separating BOIPs from their host cells to understand the effect of micronutrients directly on the pathogens is a major reason for current knowledge gaps. Of micronutrients, the role of iron has received more attention than any other, likely aided by the availability of tools, including chelators, to control pathogen access to iron. With improvements in both genetic tractability and axenic culture, including the use of chemically defined media (for *C. burnetii*) (Sandoz et al., 2016; Esquerra et al., 2017), analysis of micronutrients beyond iron is becoming increasingly feasible.

### Axenic culture and molecular genetics

Inability to physically separate (most) BOIPs from their host cells essentially prevents the analysis of pathogen responses to specific physicochemical and/or nutritional conditions. Not all isolates of *C. burnetii* can be cultured in the axenic medium ACCM-2 (Kersh et al., 2016). Understanding the metabolic basis for why *C. burnetii* isolates differ in culturability has significance both for overall understanding of metabolic capabilities and potential connections to virulence. Further exploration of how glutamine may serve as a trigger for EB-RB development and continued chlamydial replication (Rajeeve et al., 2020) may be a significant discovery on the path to designing axenic culture tools for *Chlamydia*. Axenic culture of BOIPs is especially important to allow isolation of mutants with defects in genes required for host cell invasion and/or intracellular replication, which would be selected against in systems relying on host cells for isolation and propagation. Although genetic manipulation of BOIPs remains challenging, continued progress in this area is essential for discovery and innovation in the field (Suhan et al., 1996; Rachek et al., 1998; Felsheim et al., 2010; Burkhardt et al., 2011; Kari et al., 2011; Wang et al., 2011; Beare et al., 2012; Johnson and Fisher, 2013; Noriea et al., 2015; Larson et al., 2016; Sixt and Valdivia, 2016; McClure et al., 2017; Rahnama and Fields, 2018; Ouellette et al., 2021; Fields et al., 2022; Fu et al., 2022).

## Comparative genome analysis

The following accession numbers were used to obtain genome sequence information for review of annotations or perform BLAST searches against relevant predicted proteins in *E. coli* K-12, substrain MG1655 (NC000913.3): *C. burnetii* RSA493 (NC002971.4), *C. trachomatis* L2/Bu434 (AM884176.1), *C. pneumoniae* J138 (NC002491.1), *C. muridarum* Nigg3 (NZCP009760.1), *R. prowazekii* Madrid E (NC000963.1), and *R. rickettsii* str. Sheila Smith (NC009882.1).

## Author contributions

CM: Formal analysis, Visualization, Writing – original draft, Writing – review & editing. SS: Visualization, Writing – original draft, Writing – review & editing. CM: Visualization, Writing – original draft, Writing – review & editing. WP: Funding acquisition, Visualization, Writing – original draft, Writing – review & editing. AO: Conceptualization, Formal analysis, Funding acquisition, Resources, Supervision, Visualization, Writing – original draft, Writing – review & editing.
